# ^68^Ga- or ^18^F-FAPI PET/CT—what it can and cannot

**DOI:** 10.1007/s00330-023-09715-9

**Published:** 2023-05-12

**Authors:** Yuriko Mori, Uwe Haberkorn, Frederik L. Giesel

**Affiliations:** 1 Department of Nuclear Medicine, Medical Faculty and University Hospital Duesseldorf, Heinrich-Heine-University Duesseldorf, Duesseldorf, Germany; 2 Department of Nuclear Medicine, Heidelberg University Hospital, Heidelberg, Germany

Recent innovation in nuclear medicine has led to the introduction of a novel class of ligands, named fibroblast activation protein inhibitors (FAPIs) [1–3]. In contrast to tumor entity-specific imaging strategies such as prostate-specific membrane antigen (PSMA) ligand–based PET, FAPI-PET is based on the universal approach of targeting the activated fibroblasts (cancer-associated fibroblasts: CAFs) which primarily addresses the cancer stroma (tumor microenvironment). Fibroblast activation protein (FAP) is a membrane-bound type 2 serine protease, which is overexpressed in activated fibroblasts. Through the specific binding to the enzymatic domain of FAP, FAPI-PET is able to visualize the tumor stroma formation as small as 2–3 mm [4]. In comparison to conventional PET imaging like FDG-PET, FAPI-PET is characterized by high image contrast, resulting from the low background signal in the normal organs, due to the low expression or FAP in the normal, quiescent fibroblasts.

The conjugation of quinolone-based FAP ligands with chelators such as DOTA (1,4,7,10-tetraazacylclododecane-1,4,7,10-tetrayl tetraacetic acid) enabled the radiolabeling of FAP ligands with various radiometals [1]. The gallium 68 (^68^Ga)–labeled FAPI compounds, especially ^68^Ga-FAPI-04 and ^68^Ga-FAPI-46 are currently the most widely used FAP tracers. ^68^Ge/^68^Ga-generators are commercially available and provide the possibility of a convenient small batch production in a decentralized manner. To date, multiple studies have been performed globally using the mentioned compounds, showing the potential of wide practical use, but ^68^Ga dependent PET imaging implies several limitations for broader application. The main drawback of the ^68^Ga-based imaging is the typical batch size with a maximum of 2–4 GBq for a ^68^Ge/^68^Ga generator, which allows the daily clinical performance of up to 2–3 patients per elution [5]. Another limitation is the shorter half-life of ^68^Ga (68 min), which requires an on-site and on-time synthesis of the radiotracer [6]. The delivery to the remote center is challenging. This aspect of rapid nuclear decay as well as the relatively high positron energy of ^68^Ga (1.90 MeV) should be compromised in regard to production capacity, workflow, infrastructure, and scanner quality. In a large institution with high patient throughput, several productions per day are needed to supply the demand, requiring the long attendance of qualified staff. Alternatively, the purchase of multiple generators would multiply the costs [5].

The application of ^18^F-labeled FAPI compounds provides thus an attractive option, enhancing the performance limit in the clinical routine [7, 8]. The longer half-life of ^18^F (110 min) makes the wide distribution of the ^18^F labeled tracer possible over a single metropolitan area, after the production of the compound in centers with an on-site cyclotron at a lower cost (satellite concepts) [9]. A larger batch size of several hundred GBq contributes also to the delivery of ^18^F labeled tracers to remote PET centers without a radiopharmaceutical department [6]. Regarding the image quality, the lower positron energy of ^18^F (0.65 MeV) may lead to improved spatial resolution in comparison to ^68^Ga, due to the less scattering of high-energy positrons.

The recently developed NOTA-chelator ligand FAPI-74 can be radiolabeled with both ^18^F-AIF and ^68^Ga [5]. This compound has led to the enrichment of options for tracer selection, depending on the changing local demand. The chelation of AlF was initially proposed in 2009 [10] and had been further optimized in labeling yield and specific activity. The NOTA chelator also enables theoretically chelation with ^68^Ga at room temperature, which would also allow local production in remote facilities with its own ^68^Ge/^68^Ga generator. Quality tests as required by regulatory authorities would be made easier with standardized cold kits, available for both ^18^F-AIF and ^68^Ga labeled FAPI-74. This advantage would increase flexibility for local on-demand production revolutionary, especially in institutions already equipped with ^68^Ge/^68^Ga generators and with altering clinical needs. However, the use of AlF may need careful argumentation with the legal authorities for approval.

As to the biodistributions and tracer kinetics, both ^68^Ga- and ^18^F-FAPI compounds show comparable properties, characterized by rapid renal clearance and low nonspecific uptake in normal organs. However, a recent comparison of ^68^Ga-FAPI-02, ^68^Ga-FAPI-46, and ^68^Ga-FAPI-74 revealed the highest tumor uptake and tumor-to-blood ratios for FAPI-46 [11]. These characteristics result in a lower effective dose of the FAPI tracer family in comparison with ^18^F-FDG, the current standard tracer in cancer imaging, whereby ^18^F-FAPI-74 shows a slightly lower effective dose (1.4 mSv/100 MBq) in comparison to ^68^Ga-FAPI-74 (1.6 mSv/100 MBq). Further dosimetric analysis of ^68^Ga- and ^18^F-FAPI-74 revealed a rapid time-dependent biodistribution of both tracers in tumor and normal organs, with optimal tumor-to-background ratios achieved by image acquisition 1 h after injection. The above-mentioned comparison of three tracers revealed that depending on the tracer earlier acquisitions at 30–40 min p.i. are possible depending on the tracer used [11]. In that respect, the time-dependent biodistribution in normal organs and the circulation ^68^Ga- and ^18^F-FAPI-74 showed slightly slower kinetics than other quinoline-based FAPI compounds [5, 11].

To summarize, both ^68^Ga- and ^18^F-labeled FAPI tracers have equal potential for significant contributions in clinical cancer imaging and even beyond, with comparable and favorable tracer kinetics. The selection of the tracer depends mainly upon the on-site situation, whether to prefer centralized large batch production and delivery via satellite concepts (^18^F) or decentralized smaller production but with individual concepts (^68^Ga). Both tracers enable us to gain deeper insight into the cancer behavior through stroma targeting and improved cancer staging (i.e. pancreatic adenocarcinoma, gastric cancer, hepatocellular carcinoma).

## Declarations

### Guarantor

The scientific guarantor of this publication is Frederik L Giesel.

### Statistics and biometry

No complex statistical methods were necessary for this paper.

### Study subjects or cohorts overlap

N/A
